# Evaluating the Therapeutic Effect of Dehydroepiandrosterone on Stromal Fibrosis and Endocrine Function in Patients With Resistant Ovary Syndrome: A Case-Control Study

**DOI:** 10.7759/cureus.87922

**Published:** 2025-07-14

**Authors:** Erum Gul, Shazia Sultana, Seemi Tanvir, Shama Chaudhry

**Affiliations:** 1 Department of Obstetrics and Gynaecology, Ziauddin University, Karachi, PAK; 2 Department of Pathology, Margalla Institute of Health Sciences, Rawalpindi, PAK

**Keywords:** dehydroepiandrosterone, estradiol, follicle stimulating hormone, infertility, ovarian reserve, primary ovarian insufficiency

## Abstract

Background: Resistant ovary syndrome (ROS) is a rare form of ovarian dysfunction defined by the presence of normal ovarian follicles that fail to respond to gonadotropin stimulation. The adrenal androgen, dehydroepiandrosterone (DHEA), has been studied for its potential in improving ovarian response and reserve. This study investigated the therapeutic effects of DHEA on ovarian stromal fibrosis and hormonal parameters in ROS patients.

Methods: A case-control study was conducted on 60 women diagnosed with ROS. Participants were designated as cases treated with DHEA (75 mg/day, 12 weeks) or controls (no supplementation), with 30 participants in each group. Transvaginal ultrasound (TVUS) assessments of ovarian volume, stromal fibrosis grade, and echotexture were performed before and after the treatment. Serum levels of anti-Müllerian hormone (AMH), follicle-stimulating hormone (FSH), and estradiol (E2) were also analyzed. Statistical analysis was performed using IBM SPSS Statistics for Windows, version 26 (Released 2018; IBM Corp., Armonk, New York, United States). p < 0.05 was considered significant.

Results: No significant differences were found between the two groups at baseline. By the end of 12th week, the DHEA group showed a significant improvement: 18 (60%) participants experienced a reduction in stromal fibrosis grade most commonly from Grade 2 to Grade 1, 21 (70%) showed an increase in their ovarian volume, and 14 (47%) had normalized stromal echo-texture. Changes in hormone levels were also observed, including a rise in AMH in 13 individuals (45%), a decrease in FSH in eight (28%), and an increase in estradiol in 10 (34%), which were greater than in the control group (p < 0.05).

Conclusion: Supplementation with DHEA greatly improved the ovarian stromal structure and hormonal function in ROS patients. Due to its anti-fibrotic and functional benefits, DHEA may prove to be a promising adjunctive therapy in the management of the challenging ROS condition.

## Introduction

Resistant ovary syndrome (ROS), also known as Savage syndrome, is a rare and complex cause of female infertility, in which the ovaries contain normal follicles that are incapable of responding to endogenous or exogenous doses of gonadotropins [1]. Although the follicular pool remained preserved, women with ROS frequently exhibit elevated gonadotropin concentrations, a low estrogen state, and impaired folliculogenesis, leading to anovulation and infertility [2]. The mechanism of ROS pathogenesis has been poorly defined, but is believed to comprise disrupted follicle-stimulating hormone (FSH) receptor signaling, autoimmune factors, and an unfavorable stromal microenvironment. Ovarian stromal abnormalities, specifically fibrosis, are being seen as a contributing factor in the functional resistance of ROS [3].

In this context, dehydroepiandrosterone (DHEA), a weak androgen precursor produced by the adrenal glands and ovarian theca cells, is emerging as a therapeutic agent [4]. In women with diminished ovarian reserve (DOR), it has been shown that DHEA exerts multiple physiological benefits on ovarian function, including improved follicular development, oocyte quality, and increased responsiveness to gonadotropins [5]. Due to its anti-fibrotic and stromal remodeling properties, it is considered an attractive candidate to counteract the ROS, especially when standard gonadotropin therapy frequently fails [6,7]. Fibrosis of the ovarian stroma may cause localized disruption of the microvascular network and the paracrine signaling necessary for normal folliculogenesis. Although many studies suggest that DHEA can help improve ovarian reserve [8], its role in treating stromal fibrosis in women with ROS is still unestablished.

The objective of this study was to evaluate the potential of DHEA to reverse or prevent ovarian resistance related to fibrosis. The therapeutic implications of DHEA supplementation on ROS patients, concerning ovarian stromal fibrosis, hormone profiles, and morphological parameters, were also explored in this study, thereby offering new insights into adjunctive ROS treatment strategies. The results of this study may suggest the possibility of refining fertility interventions and provide support for the development of individualized strategies for women suffering from this rare and debilitating disorder.

## Materials and methods

This was a case-control study conducted over three months (December 2024 to March 2025) at Dr. Ziauddin Hospital, Karachi, Pakistan. The study was approved by Dr. Ziauddin Hospital (approval number: 14/3/25/1425MS), and was conducted in accordance with the Declaration of Helsinki. All participants provided written informed consent before enrollment.

Eligibility

Women aged 25-38 years with a confirmed diagnosis of ROS, a normal uterine cavity on transvaginal ultrasound, and a baseline anti-Müllerian hormone (AMH) level <1.2 ng/mL or an antral follicle count (AFC) <5 were included in the study. Clinical diagnosis of ROS was based on criteria of normal antral follicle count on ultrasound, elevated serum gonadotropin levels, and lack of response to exogenous gonadotropin stimulation despite adequate ovarian reserve markers. Exclusion criteria included polycystic ovary syndrome (PCOS), endometriosis, or history of ovarian surgery, autoimmune or endocrine disorders, or use of hormonal therapy within the past three months. 

Sample size calculation

The sample size was calculated using OpenEpi version 3.0.0 [9] for 80% power with an alpha of 0.05, requiring 30 participants per group to meet the minimum requirement [10]. A simple random sampling technique was used to randomly assign participants to two equally sized groups: a DHEA group (n = 30) who took the DHEA supplementation at 75 mg/day orally for 12 weeks, and a control group (n = 30) who received no supplementation.

Data collection

Baseline characteristics were measured, including transvaginal ultrasound (TVUS) for ovarian volume, stromal fibrosis grading, and stromal echo-texture. In the blood serum, AMH, FSH, and estradiol (E2) were assessed using standardized immunoassays. Grading of stromal fibrosis was qualitatively performed using standardized ultrasound criteria: Grade 2 indicated moderate fibrosis, and Grade 1 indicated mild (or reduced) fibrosis. Twelve weeks later, the same assessments were repeated. In the early follicular phase (2-5 days), a high-resolution transvaginal probe (7.5 MHZ) was used for the ultrasound. Fasting blood samples were processed in the same certified laboratory to obtain hormonal profiles. Compliance with DHEA supplementation was determined by participants' monthly pill counts.

Data analysis

Data were analyzed using IBM SPSS Statistics for Windows, version 26 (Released 2018; IBM Corp., Armonk, New York, United States). Age and BMI were continuous variables and presented as mean ± standard deviation (SD) and were compared using independent sample t-tests. Stromal fibrosis, increase in ovarian volume, and hormonal improvement percentages were expressed as counts and percentages and compared using Chi-square tests. Statistical significance was set at p < 0.05.

## Results

A total of 60 women with ROS were recruited and assigned equally to the DHEA (n = 30) and control (n = 30) groups. The groups had similar baseline features. By the end of 12 weeks, the DHEA group showed decreased stromal fibrosis, a larger ovarian size, and improved stromal appearance. Hormone profile testing revealed that AMH and E2 were increased, whereas FSH level was reduced, suggesting improved ovarian function. Table 1 displays the baseline characteristics of patients in both groups.

**Table 1:** Baseline patient characteristics DHEA: dehydroepiandrosterone; BMI: body mass index; AMH: anti-Müllerian hormone; AFC: antral follicle count

Characteristic	DHEA Group (n=30)	Control Group (n=30)	Test Used	Test Statistic	p-value
Age (years), mean±SD	32 ± 3.1	31 ± 3.4	Independent t-test	t = 1.17	0.24
BMI (kg/m^2^), mean±SD	24.6 ± 1.2	24.5 ± 1.3	Independent t-test	t = 0.31	0.76
Low AMH, n (%)	27 (90%)	26 (88%)	Chi-square test	χ² = 0.10	0.75
AFC < 5, n (%)	28 (93%)	27 (90%)	Chi-square test	χ² = 0.21	0.65

Age, BMI, and indicators of baseline ovarian reserve were similar in both groups. Both had low AMH (n=27 (90%) versus n=26 (88%)) and an AFC <5 (n=28 (93%) versus n=27 (90%)) in the DHEA and control group, respectively. Both groups had matched ovarian dysfunction at the start. Through these characteristics, DHEA was offered a uniform base for evaluation. Table 2 shows the values for morphological improvements in the ovary and fibrosis.

**Table 2:** Ovarian morphology and fibrosis Improvement * statistically significant (p < 0.05) DHEA: dehydroepiandrosterone; ↓: decrease; ↑: increase

Ovarian Improvement	DHEA Group (n=30), n (%)	Control Group (n=30), n (%)	Test Used	Test Statistic	p-value*
↓ Stromal Fibrosis (Grade 2→1)	18 (60%)	5 (15%)	Chi-square test	χ² = 12.86	<0.001
↑ Ovarian Volume	21 (70%)	6 (20%)	Chi-square test	χ² = 15.60	<0.001
Normal Stromal Echo-Texture	14 (47%)	4 (13%)	Chi-square test	χ² = 8.31	0.004

DHEA dramatically decreased ovarian stromal fibrosis in 18 participants (60%) and improved visual appearance. Increased ovarian volume was detected in 21 individuals (70%) by ultrasound in the treatment group, and 14 (47%) DHEA users achieved normalization of stromal texture. These results showed the structural improvements with DHEA therapy. Table 3 highlights the hormonal profile after 12 weeks of DHEA administration.

**Table 3:** Hormonal profile after 12 weeks *statistically significant (p < 0.05) DHEA: dehydroepiandrosterone; AMH: anti-Müllerian hormone; FSH: follicle-stimulating hormone; ↑: increase; ↓: decrease

Hormone Response	DHEA Group (n=30), n (%)	Control Group (n=30), n (%)	Test Used	Test Statistic	p-value*
↑ AMH Levels	13 (45%)	2 (8%)	Chi-square test	χ² = 11.09	0.001
↓ FSH Levels	8 (28%)	2 (7%)	Chi-square test	χ² = 5.05	0.025
↑ Estradiol (E2)	10 (34%)	3 (10%)	Chi-square test	χ² = 5.65	0.017

Serum AMH, a marker of ovarian reserve, was improved by DHEA intervention in 13 study subjects (45%). Almost one-third of the treated group, including eight participants (28%), experienced reduced ovarian stress with decreased FSH levels. Ten participants (34%) demonstrated a rise in E2, suggesting better ovarian activity. These findings also propose that hormonal changes obtained through DHEA play a role in enhancing ovarian function.

## Discussion

In this study, women diagnosed with ROS were treated with DHEA to evaluate its therapeutic effect on ovarian stromal fibrosis and endocrine function. Participants showed substantial improvement in ovarian morphology and hormonal profile after 12 weeks of oral DHEA supplementation. These findings highlighted the therapeutic potential of DHEA to target the underlying pathophysiology of ROS. Transvaginal ultrasound demonstrated a significant reduction in fibrosis and restoration of stromal architecture, implying that DHEA may reverse fibrotic changes in the ovarian stroma. Because fibrosis may serve as a mechanical and biochemical barrier to follicular development, reversing fibrosis may improve ovarian responsiveness [10].

Our results are in line with the previously published studies regarding DHEA's effect on boosting ovarian function in patients with depleted ovarian reserves. It was shown that DHEA improved ovarian response during assisted reproductive treatment in poor responders [11]. Furthermore, other studies also demonstrated the stimulation of follicular development and oocyte yield in women supplemented with DHEA [12]. The observed effects are presumed to occur through androgen receptor pathways, which promote folliculogenesis by enhancing granulosa cell proliferation and upregulating FSH receptors [13]. However, DHEA also has androgenic anti-fibrotic actions via cytokine modulation, particularly by transforming growth factor-beta (TGF ß) downregulation that is a key mediator of tissue fibrosis [14,15].

Studies in other fibrotic tissues have revealed that DHEA suppresses collagen synthesis and inhibits the accumulation of extracellular matrix through the PI3K/AKT signaling pathway [16]. In the ovarian context, this could provide a more favorable stromal environment to favor follicle survival and maturation. DHEA is a noninvasive, accessible, and low-risk adjunctive treatment for women with ROS, an often-refractory condition where current treatments rarely yield an adequate response [17]. DHEA may recover both the structural and functional aspects of the ovary, consequently improving natural ovulation and increasing success rates in fertility treatments. In addition, it has a favorable safety profile and can be administered orally, which supports its applicability in routine clinical practice [18].

Nevertheless, this study had several limitations. Due to the design of the observational approach, causality was not conclusively determined. Although participants were randomized, the absence of blinding introduced the risk of bias. Ultrasound imaging, rather than a histological confirmation, was used to assess fibrosis. In addition, pregnancy or live birth rates were not evaluated. Randomized, placebo-controlled trials with histopathological correlation of stromal changes, longer follow-up for reproductive endpoints, and studies of optimal dose and duration of DHEA therapy should be conducted in the future.

## Conclusions

DHEA supplementation for 12 weeks showed significant improvements in the appearance of the ovaries and their hormone levels in women with ROS. The reduced fibrosis in the stroma and an increased ovarian size were observed structurally, subsequently followed by improved hormonal profile. These results imply that DHEA could prevent stromal changes and increase the function of the ovaries.

This study demonstrated that DHEA might be useful in addressing ROS, especially when regular treatments fail to respond. Since it is given orally and has a good safety record, it is used in clinical settings. Further studies on the investigation of multiple variables should be conducted to validate these results and observe long-term reproductive effects.
